# How to Prevent Suicide in Older Patients with a Neurocognitive Disorder: A Scoping Review Leading to the Development of a Clinical Guide for Healthcare Workers

**DOI:** 10.3390/healthcare14010036

**Published:** 2025-12-23

**Authors:** Sylvie Lapierre, Cécile Bardon, Charles Viau-Quesnel, Jean Vézina, Rock-André Blondin, Catherine Gagnon, Isabelle Lafleur, Christophe Marchand-Pellerin, Myriam Gauvreau, Nicole Poirier

**Affiliations:** 1 Centre for Research and Intervention on Suicide, Ethical Issues and End-of-Life Practices, University of Quebec in Montreal, Montréal, QC H3C 3P8, Canada; bardon.cecile@uqam.ca; 2 Department of Psychology, Université du Québec à Trois-Rivières, Trois-Rivières, QC G9A5H7, Canada; charles.viau-quesnel@uqtr.ca (C.V.-Q.); christophe.marchand-pellerin@uqtr.ca (C.M.-P.); myriam_gauvreau@hotmail.com (M.G.); 3 Department of Psychology, Laval University, Québec, QC G1V 0A6, Canada; jean.vezina@psy.ulaval.ca; 4 Montfort Hospital, Ottawa, ON K1K 0T2, Canada; rockablondin@montfort.on.ca; 5 Department of Psychology, Faculty of Social Sciences, Ottawa University, Ottawa, ON K1N 6N5, Canada; 6 Integrated Health and Social Services Centres (CISSS) des Laurentides, Saint-Jérôme, QC J7Z 4M2, Canada; catherine.gagnon.cissslau@ssss.gouv.qc.ca (C.G.); isabelle.lafleur.lddm@ssss.gouv.qc.ca (I.L.); 7 Carpe Diem House, Trois-Rivières, QC G8Z 2N7, Canada; npoirier@carpediem.quebec

**Keywords:** suicide, neurocognitive disorders, prevention, intervention, long-term care, geriatric settings

## Abstract

Background/Objective: Healthcare professionals working with individuals living with neurocognitive disorders (NCD) express the need for training to prevent suicidal behaviors in this population. Accordingly, this paper describes the process used to develop a suicide prevention clinical guide for use in geriatric care settings. Methods: The project involved three steps. First, a team of researchers conducted a scoping review of empirical studies on suicide among older adults with NCD, focusing on prevalence, risk and protective factors, assessment and practical interventions. Secondly, based on these findings, the team created a clinical guide that helps healthcare professionals assess needs and suicide risk and formulate action plans to improve well-being, ensure safety, and reduce the risk of suicide. Result: The guide was finalized after 18 months of deliberation. It enables professionals to structure their evaluation, so that no relevant aspect is overlooked, and protective factors are reinforced. It emphasizes shared responsibilities and interdisciplinary collaboration. It recommends that professionals conduct a personalized clinical assessment of unmet needs to reduce distress. During the third step, the guide was evaluated through a pilot study, involving post-training focus groups and interviews with professionals who used it in clinical practice. Conclusions: Participants’ feedback was integrated into the final version of the Guide, and the results indicated that it helped dispel misconceptions about the low risk of suicide among patients with NCD, whose suicidality is frequently misinterpreted as mere disruptive behavior. Organizational barriers represent the main challenge professionals may face when using the Guide.

## 1. Introduction

In 2018, an estimated 55 million people worldwide were living with major neurocognitive disorders (NCD), and this number is projected to triple to 152 million by 2050 [1,2]. Therefore, the increasing prevalence of NCD, which doubles every 5 years from the age of 65 [3], constitutes a major challenge for the healthcare and social services system. This upward trend in dementia prevalence reflects the accelerated aging of the global population and rising life expectancy.

In the absence of curative treatment, the announcement of an NCD diagnosis triggers several reactions such as shock, denial, negative emotions (e.g., anger, helplessness, insecurity, shame, frustration), or withdrawal from social life [4], but also prompts individuals to use various coping strategies to preserve their identity and maintain hope, quality of life, and a sense of control [5]. Some individuals can experience depression and anxiety following the diagnosis [6], and for those with preserved insight, awareness of cognitive decline and fear about the future may worsen these symptoms and heighten the risk of suicidal thoughts and behaviors [7]. Healthcare workers often feel ill-equipped to address these emotions [8,9].

In the fall of 2021, professional practice advisors from an Integrated Health and Social Services Centre (IHSSC) contacted one of the authors, an expert on suicide in older adults, to request suicide screening tools and prevention protocols for individuals with NCD living in long-term care (LTC) facilities. A scientific team was created to answer this request. It comprised specialized researchers in gerontology, suicide prevention, and NCD, as well as experienced geriatric healthcare professionals and service organization specialists of the IHSSC, who manage geriatric care at home and in residential settings for seniors with NCD. The goal of the research project included three steps. Given the emerging and complex nature of this topic, the first step involved a scoping review of research on suicide among individuals with NCD. The second step aimed to develop a clinical guide for suicide risk assessment and prevention, based on an analysis of the current literature and the team’s expertise. Finally, in the third step, the guide and its tools were evaluated through a pilot study with IHSSC healthcare professionals. This evaluation assessed the guide’s conceptual structure and its preliminary feasibility and implementation in clinical settings. The results of the pilot study were incorporated in the final version of the Guide presented here.

## 2. Scoping Review

### 2.1. Method

This scoping review was conducted following the Preferred Reporting Items for Systematic Reviews and Meta-Analyses extension for Scoping Reviews (PRISMA-ScR) guidelines. No protocol was developed or registered because this scoping review was intended as an exploratory mapping of the literature rather than a systematic review with narrowly defined outcomes.

Studies were eligible for inclusion if they were empirical, peer-reviewed publications from the year 2000 onward, examining suicidality among older adults with NCD. Suicidality was defined broadly to encompass a wish to die, suicidal ideation, suicidal behavior, suicide attempts, and death by suicide. Eligible studies addressed at least one of the following outcomes: prevalence of suicidality, associated risk or protective factors, suicide risk assessment, or prevention and intervention strategies aimed at improving well-being and reducing suicidality. The literature search was conducted in two electronic databases, Medline and PsycINFO, in English or French (all authors are fluent in French), using the following keywords in the subject field (SU): dementia OR neurocognitive disorders AND suicid*.

**Figure 1:**
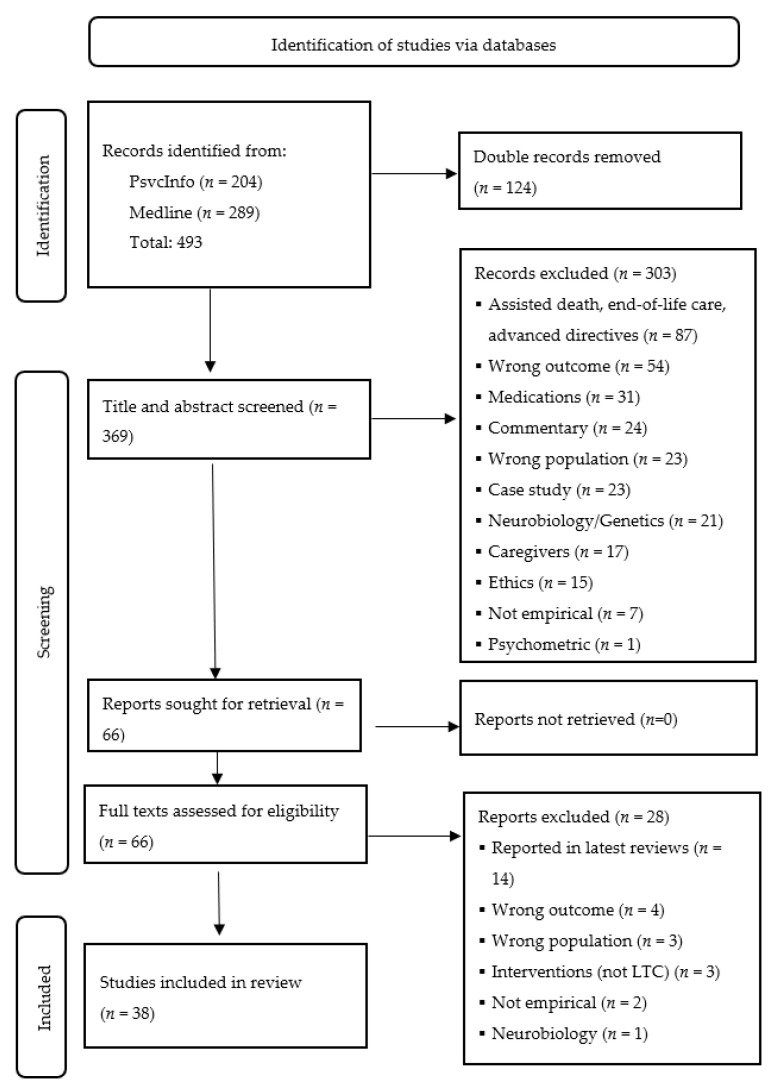
PRISMA flow diagram for the scoping review.

The study selection process is presented in the PRISMA flow diagram (Figure 1). The database search yielded 493 records (Medline = 289; PsycINFO = 204). After removing 124 duplicates, 369 titles and abstracts were screened independently for relevance by two reviewers using the pre-mentioned eligibility criteria.

Studies on assisted death, end-of-life care, ethics, and advance directives were excluded. Although some jurisdictions (e.g., Belgium, the Netherlands, and Quebec in Canada) allow individuals with a diagnosis of NCD to make an advance directive for medically assisted death, the request must be made while the person still has decision-making capacity. The directive is intended to be implemented later, once that capacity is lost and the specified clinical symptoms occur. For healthcare professionals, assisted death has introduced significant complexity and ethical tension in clinical decision-making related to the management of the patient’s situation and respect for their autonomy. Nonetheless, professionals are still required to intervene when a person is suicidal and when no advance care plan has been made. Moreover, rate of completion of advance care planning among older adults with NCD is low [10]. Also, studies focusing on informal and formal caregivers’ burden, as well as physiological and pharmacological aspects of NCD, were excluded. In fact, while numerous factors can lead to the development of an NCD, allied health professionals who are in daily contact with individuals with NCD are unable to address the cause of the disease. Since the primary goal of the project was to develop training for healthcare workers to assess patients’ distress and suicide risk, while providing supportive care to manage symptoms and improve quality of life, these topics were excluded.

Among the 369 reports identified, 66 full-text articles were assessed for eligibility, with 38 studies included in the final analysis. Priority was given to population-based studies, along with systematic reviews and meta-analyses that often describe findings from earlier studies on the topic. Key information was extracted to summarize the existing literature on prevalence, risk predictors, and suicide prevention strategies among older adults with NCD.

### 2.2. Dementia Diagnosis and Suicidal Ideation

While some individuals experience heightened depression and anxiety following a diagnosis of NCD [6,11,12], research has also reported the presence of suicidal ideation and behavior in this population [13,14,15,16,17,18,19,20]. In fact, a recent meta-analysis by Medeisyte et al. [21] showed that individuals with NCD and comorbid depression were at significantly higher risk of suicidality, including suicidal ideation (OR = 5.11, 95% CI [1.73; 15.07]), attempt (OR = 7.75, 95% CI [2.68; 22.41]), and death (OR = 3.44 95% CI [1.65; 7.18]). Men with dementia were more likely to attempt (OR = 1.28, 95% CI [1.25; 1.31]) and die by suicide (OR = 2.88, 95% CI [1.54; 5.39]) than women with dementia [22]. Regarding prevalence, a study conducted in eight European countries, among community-dwelling adults aged 65 and older with NCD, revealed that the frequency of suicidal ideation varied between 6% and 24% [18]. Furthermore, a recent meta-analysis indicated that the prevalence of suicidal ideation was higher among individuals with NCD (10%) than the general population (2%) [22].

The four most relevant studies on prevalence and risk factors have relied on national centralized population registers [23]. For example, over a 10-year period (2007–2017), Hedna et al. [24] identified 59,042 Swedes aged 75 and older who had been diagnosed with dementia. They found that the rate of suicidal behavior was 53 per 100,000 person-years for the entire cohort (65 in men and 47 in women), while the rate of suicide was 12 per 100,000 person-years (18 in men and 8 in women).The study showed that the adjusted risk ratio for suicidal behavior was higher in individuals with past episodes of self-harm, those with severe depression, and users of anxiolytic or hypnotic medications, as well as in individuals with mild cognitive decline (MMSE > 20) or a high frailty score (assessed during the 5 years preceding the diagnosis). Results also showed that the adjusted risk ratio was lower in people aged 85 and older, as well as in those receiving home care. For suicide, the predictors were in the same direction, although the effects were smaller. The authors consider that home care serves as a protective factor because it allows individuals with NCD to remain in a familiar environment, reducing loneliness, preventing destabilizing changes, and facilitating the communication of relevant clinical information to healthcare professionals to ensure optimal medication management. It is also interesting to note that the risk estimates for suicidal behaviors were similar, regardless of the type of dementia. However, this study only included people with a clinical diagnosis of major NCD, and it is possible that these findings may not apply to those with preclinical conditions [25].

In Denmark, Erlangsen et al. [26] conducted a retrospective (1980–2016) nationwide cohort study of all individuals aged 15 years and older (N = 7,300,395) to investigate whether there was an association between dementia and other neurological disorders (e.g., amyotrophic lateral sclerosis, stroke, etc.) and a higher risk of death by suicide. Results for dementia showed that the incidence rate ratio (IRR) of suicide was lower (0.8, 95% CI [0.7–0.9]) than for other neurological disorders. However, the risk was almost four times higher during the first month after diagnosis (IRR = 3.0, 95% CI [1.9–4.6]), highlighting the distress felt by individuals when faced with this news. Therefore, the period surrounding the diagnosis is a critical moment for intervention. Furthermore, Erlangsen et al. [16] observed that individuals diagnosed with dementia at an unexpected age, that is, between 50 and 69 years, had higher suicide rates compared to those diagnosed at 70 years or older. Erlangsen [23] also notes that individuals in the early or mild stages of the disease are at higher risk of suicidal behavior than those in advanced stages, with risk decreasing over time, 1–2 years (0.7, 95% CI [0.5–0.9]) and 3–5 years (0.5, 95% CI [0.4–0.7]), suggesting that disease progression may reduce the ability to plan or carry out the suicidal act. Holmstrand et al. [18] and Ortner et al. [27] reported similar findings: patients who expressed suicidal ideation during early stages of dementia often stopped expressing them in advanced stages. However, it remains unclear whether this was due to reduced communication abilities, a reduction in disease awareness, and/or an adjustment to their situation.

Using South Korean population databases, Goh and Park [28] conducted a case–control study of 80,791 cases of suicide from 2012 to 2017, matched with living individuals of the same age and sex. They found that a diagnosis of major NCD within one year before death was associated with a threefold higher risk of suicide. In contrast, risk diminished when diagnoses were made two to five years prior. These observations support Choi et al. [29], who found in a cohort of 36,541 South Koreans aged 60 and older that suicide risk increases during the year following a dementia diagnosis compared to those without the diagnosis.

In the U.S., Schmutte et al. [30] studied the records of 2.667 million Americans aged 65 and older who were diagnosed with dementia between 2012 and 2015 to examine the risk of suicide in the first year after diagnosis. Researchers found a 12-month suicide rate of 26.42 per 100,000 person-years for this cohort. Individuals with a diagnosis of frontotemporal dementia were also found to have a higher risk of death by suicide (rate of 124.6 per 100,000 person-years) compared to 23.74 for Alzheimer’s disease. Lai et al. [31] and Zucca et al. [32] made the same observation for this subtype of dementia (for a review on dementia subtypes and suicidality, see [33]). Compared with the general population, older adults diagnosed with dementia were at 1.53 times higher risk of suicide, but this risk was even higher among those aged 65 to 74 (3.40). Like other researchers, Schmutte et al. [30] also found that the risk decreased with age. Interestingly, most suicide deaths occurred within the first 90 days after diagnosis (41.7% in women and 48.4% in men). In a population-based longitudinal study of American veterans (N = 147,595), Günak et al. [17] found that the risk of suicide attempts was higher among those recently diagnosed with mild or major NCD, even after adjusting for demographic characteristics as well as medical and psychiatric comorbidities. Annor et al. [34] made similar observations.

Finally, in a population-based case–control study in England (N = 594,674), Alothman et al. [35] found no overall significant association between a major NCD diagnosis and suicide risk (adjusted OR, 1.05; 95% CI [0.85–1.29]). However, suicide risk was 6.69 times (95% CI, 1.49–30.12) higher in patients younger than 65 years and within three months of diagnosis compared to patients without dementia.

### 2.3. Suicidal Ideation Predictors

Holmstrand et al. [18] sought to identify factors that statistically predicted suicidal ideation in people with NCD. Bivariate analyses showed that the associated variables were depressive symptoms, delusional symptoms, agitation, anxiety, apathy, disinhibition, irritability, nighttime behavioral disturbances, and taking anxiolytic or dementia medications. It therefore appears that people with behavioral and psychological symptoms of dementia (BPSD) are at higher risk of suicidal ideation (see also [27,36]. In the regression analysis, the variables significantly associated (*p* < 0.05) with suicidal ideation were a moderate stage of cognitive disorder as assessed by the standardized Mini Mental State Exam, depressive and delusional symptoms, and anti-dementia medication. Other researchers report that psychiatric comorbidities and/or previous suicide behaviors increased the risk for suicidality in people with NCD [17,29,30,31,35,37,38,39]. In addition, Rymo et al. [40] reported that both past year and lifetime life-weariness were more frequent among individuals with mild impairment than among those who were cognitively intact.

There are several possible explanations for these observations. Among individuals with preserved insight, awareness of the disease and its progression may heighten feelings of despair [37,41]. Also, many NCDs can involve disinhibition or impulsivity, which increases the risk of suicidal behavior [37,42]. This impulsivity may be associated with a reduced problem-solving ability, especially in depressed older adults, potentially increasing the risk of considering suicide as a solution to current and anticipated difficulties [43]. Social isolation, as well as perception and fear of being a burden to loved ones, can further increase the risk of suicidal behavior [44,45,46]. However, these suicide risk factors, along with other potential confounders such as life events and unmet needs (inability to engage in meaningful activities and social connections), as well as altruistic suicide to protect next of kin [47], have not yet been seriously investigated in dementia [13,37].

It is generally thought that individuals with major NCD are incapable of planning and attempting suicide because of impaired executive function. Although advanced NCD might have a protective effect against suicide due to the gradual loss of the executive abilities [48], various preserved cognitive functions, physical abilities, and impulsivity can maintain the capacity to end one’s life, which may explain the higher prevalence of suicidality in the mild to moderate stages of the disease [15,16,17,20,24,34,49,50].

### 2.4. Suicide in Long-Term Care Facilities

According to the systematic review by Joshaghani et al. [51], 12 studies have investigated, since 2010, the suicide risk among patients aged 65 years and older with dementia residing in LTC facilities. All studies indicate that the risk of suicide is lower in LTC facilities, partly due to the constant presence of staff and the limited access to means of self-harm. Although suicide deaths are rare, residents often express both passive (wish to be dead) and active (desire to end one’s life) suicidal ideation, as well as direct (pinching, biting, hitting, cutting oneself, pulling own hair, banging one’s fist against objects) and indirect (resistance to care, refusal to drink or eat, spitting out or refusing medication) self-injurious behavior [52,53,54,55]. In a meta-analysis of data from 3,023,224 individuals aged 60 and older living in LTC institutions, the prevalence of self-harm behaviors, regardless of intent, was estimated at 40.4%, suicidal ideation at 12%, and suicidal behaviors at 6.4% [56]. However, although most seniors living in LTC facilities have an NCD, Joshaghani et al. [51] and Bareeqa et al. [56] did not provide prevalence estimates according to this condition.

On the other hand, Joshaghani et al. [51] identified several suicide risk factors in individuals with NCD living in LTC facilities: younger age (65–74 years), newly diagnosed NCD (less than 1 year), mild NCD, comorbid depression or anxiety, past suicidal behavior, presence of pain and sleep problems, and transition or admission to an LTC facility. Protective factors include older age, severe NCD with impaired insight, physical limitations preventing the execution of a suicide plan, and positive social relationships and frequent visits from loved ones, as well as increased monitoring by mental health professionals [51].

### 2.5. Intervention with Suicidal Patients in Long-Term Care Facilities

Despite the elevated risk of suicidality among individuals with NCD, specific interventions or care guidelines remain rare, even though many authors recommend systematic suicide risk screening at admission and after significant losses, along with the development of preventive therapeutic and behavioral interventions [22,24,45,51,57]. Only a handful of studies have looked at suicide prevention in LTC facilities, but the efficiency of the intervention is largely unknown [54,58,59,60,61]. Furthermore, almost no studies have examined interventions specifically tailored for residents with NCD.

Clinical practice typically emphasizes the management of BPSD, often overlooking the socio-emotional well-being and quality of life of patients [22]. However, some psychosocial interventions have sought to improve quality of life in LTC facilities by reducing BPSD associated with boredom, loneliness or sensory deprivation, using gardening, sensory stimulation, or various physical and meaningful activities [62]. Other interventions use music [63,64] or the snoezelen method (controlled multisensory stimulation promoting relaxation) for people with major NCD [65].

In the city of Trois-Rivieres (Canada), an innovative, private, small LTC facility was established in 1995 by Ms. Nicole Poirier for individuals with NCD, adopting a unique humanistic approach to the illness [66,67]. Called Carpe Diem (a Latin word meaning “Seize the day” or “Make the most of this day”), the organization and services are tailored and personalized to meet each individual’s needs and help to maximize autonomy. Instead of focusing on deficits, the intervention concentrates on the development and preservation of the person’s remaining abilities and resources. The approach is based on a trusting relationship in which the person feels accepted, and on activities (such as domestic chores, gardening, sports, arts and music workshops, cultural outings) that promote personal fulfilment, fostering feeling of contribution and usefulness [67,68]. All behaviors are considered meaningful and convey messages addressed to family or professional caregivers who must strive to understand them. Medication is not used to control the person, nor is it intended to replace human assistance or compensate for organizational gaps. The approach has been in use for 30 years, with training extended to numerous organizations across Francophone countries. In a pre-post training study of the Carpe Diem approach implemented in an LTC institution in France, using the *Neuropsychiatric Inventory* (NPI)—a caregiver-based interview covering 12 common BPSD [69]—results showed significant reduction in agitation, apathy, irritability, sleeping difficulties, and eating behavior disorders among patients with NCD [70]. This type of personalized approach appears to enhance the well-being of individuals with NCD and was incorporated into the clinical guide to address unmet needs and prevent suicidality [13].

### 2.6. Suicide Risk Assessment

Although several studies emphasize the need to adapt suicide risk assessment tools to the progression of the NCD [20,71], existing tools are unsuitable and inappropriate for people with this illness [72]. Similarly, after more than 30 years of research on suicide, Hawton et al. [73] note that scaled-based approaches to clinical risk assessment are ineffective to predict suicide and that all individuals (with or without dementia) are potentially at risk of ending their lives. They recommend that professionals conduct a clinical assessment of unmet needs, identify modifiable risk factors, and suggest changes to enhance quality of life and well-being, followed by a personalized action plan to ensure the person’s safety. These recommendations seem appropriate for intervening with older adults with NCD who have suicidal ideation or behavior.

### 2.7. Conclusions and Limitations of the Scoping Review

The scoping review found that suicidality (including suicidal ideation, attempts, and death) is highly prevalent among individuals with NCD, particularly those with comorbid depression, a recent diagnosis, or mild to moderate cognitive decline. Although suicide risk among patients with NCD is lower in LTC facilities, distressed residents may still engage in self-injurious behavior and express a wish to die. In this context, healthcare workers need training to better prevent suicidality in their patients. However, the scoping review also found that existing suicide risk assessment tools are not suited for this population, and that tailored interventions or care guidelines remain very scarce, justifying the need for the development of a clinical tool specifically designed to assess and manage suicide risk in this group. It should be mentioned that this scoping review has a few limitations. Since it was intended as an exploratory mapping of the literature on the issue, there is no registered protocol nor any quality assessment of the included studies. Moreover, the selected studies show considerable heterogeneity in population, especially in NCD subtypes and disease stages, as well as in suicidality outcomes. Future reviews should examine the grey literature—including theses, conference proceedings, and reports—to identify current interventions aimed at reducing suicidality among individuals with NCD.

## 3. Development of a Clinical Guide to Prevent Suicide

Building on the scoping review findings, a steering committee of experts in psychogeriatry and suicide prevention proceeded to the second phase of the project to develop a clinical guide supporting healthcare professionals in assessing the needs and suicide risk of patients with NCD and identifying ways to reduce suicidal behaviors. The process was conducted in collaboration with the IHSSC Administrative Division of Programs Supporting Older Adults’ Autonomy, which manages geriatric services at home, as well as in intermediate resources and private residences housing seniors with NCD. Following the recommendation of Portacolone et al. [45], the creation of the guide relied on Skivington et al. [74]’s *Framework for Developing and Evaluating Complex Interventions*. The process, conducted over 18 months through six meetings, integrated current theoretical and empirical evidence (e.g., risk and protective factors of suicide in older adults, particularities of suicide in individuals with NCD), and considered contextual factors (e.g., characteristics and symptoms of patients, role of the health system in the issue, and barriers to the provision of appropriate treatment). The meetings also addressed key uncertainties (e.g., when, how, and who to screen for suicide risk), considering the availability of services and human resources, and ensuring that the intervention is continuously refined throughout its implementation. The process actively involved geriatric care workers who described specific cases, highlighting the challenges they faced in identifying and managing suicide risk among patients with NCD. The professional practice advisors at IHSSC planned and managed the meetings and workflow, publishing the first edition of the Guide [75].

During the third step, the Guide was evaluated during a pilot study that involved two post-training focus groups with eight health practitioners (*n* = 16), and follow-up individual interviews with six participants who used the guide in their clinical practice over a 5-month period [76]. Feedback from participants was integrated in the second edition of the Guide [77], using IHSSC stakeholders’ organizational processes and terminology to enhance adaptation and acceptability. The results of the pilot study are embedded in the following description of the final version of the Guide [76].

### 3.1. General Structure and Aim of the Guide

The Guide was primarily intended for professionals (e.g., nurse, social worker, special education technician, physiotherapist, occupational therapist) working in intermediate resources, private seniors’ residences, hospitals and geriatric services (e.g., home care). However, it can be used by anyone interacting with older adults with NCD.

The final 46-page guide, called *Intervening with individuals with a neurocognitive disorder (NCD) and at risk of suicide*, aimed to support care professionals in carrying out a personalized clinical assessment of suicide risk and formulate action plans aimed at enhancing patients’ well-being and thereby reducing the risk of suicide among individuals with NCD [77].

The guide provides an in-depth, person-centered analysis of the situation to address unmet needs and help reduce distress [13,78]. It integrates, in a single document, a synthesis of the available information on suicide risk and protective factors in older adults (e.g., [79,80]); BPSD, including agitation, irritability, and decline in communication skills [81]; and the influence on well-being of the approach used with the patient as well the characteristics of the physical and social environment in which they live (e.g., [82,83]).

The Guide does not provide a standardized assessment scale, nor does it categorize the level of risk for a suicide attempt or predict suicide behaviors. Rather, it explains the process for the clinical analysis of the situation, grounded in a humanistic approach for individuals with NCD [67] and informed by previous suicide interventions with children aged 5 to 13 [84] and individuals presenting specific cognitive vulnerabilities, such as intellectual disabilities [85].

The clinical process is structured into five major steps to support analysis and clinical judgment: Identify, Screen, Assess, Plan the intervention, and Re-assess the situation. This process begins with the observation of the patient in their own environment. The Guide allows professionals to structure their thoughts, organize the interview with the person in distress, and ensure that no element relevant to the assessment is overlooked (e.g., presence of a suicide plan). It also encourages them to be attentive to nonverbal manifestations of distress and symbolic gestures, while reassuring them about the importance of openly discussing suicide with the patient.

The Guide emphasizes the importance of shared responsibilities and interdisciplinary collaboration. Thus, all staff members are trained to recognize signs of distress, identify individuals with suicidal ideation and take appropriate action in the situation. Collaboration and mutual support help to break the isolation and helplessness felt by some professionals when faced with older adults’ suicidal behavior. This approach also prevents delays in suicide risk assessment, which is usually conducted only after a referral to psychosocial professionals, while allowing for effective prioritization that ensures that cases are handled at the right time. Nonetheless, participants in the pilot evaluation of the program felt that a professional who had already established a therapeutic alliance with the person with NCD would be better able to interact with them and obtain the information necessary to assess the risk of a suicide attempt [76]. The Guide also encourages professionals to involve family members in sharing information about the person with NCD, such as life history and preferences. Family input helps professionals understand the patient’s behavior, assess distress, and develop a safety plan, while ensuring a warm presence and ongoing monitoring. The goal of the clinical process is to identify and take appropriate short- and medium-term actions to address emotional distress and unmet needs to reduce suicidal ideation and behaviors. The Guide also recommends assessing and monitoring the impact of these interventions and strategies on the patients’ well-being. It is important to note that the Guide does not replace the strategies outlined by the organization’s suicide policy and procedures; rather, it complements them by addressing the specific needs of individuals with NCD. Indeed, the standard suicide prevention procedure (three pre-established screening questions: Are you thinking about suicide presently? Have you thought about suicide in the last months? Have you attempted suicide in the last year?) can be applied when the person is at the early stage of the illness and their cognitive functions seem relatively preserved.

Finally, the Guide provided various tools that healthcare workers could use according to their needs: (1) a checklist summarizing key elements to consider at each step of the process; (2) a form titled *Assessing the Risk of Suicidal Behavior in a Person with NCD*, which highlights critical factors that support systematic analysis and inform clinical judgment; (3) a form titled *Safety Plan for Addressing Suicidal Ideation in a Person with NCD*; (4) a note-taking template for documenting information in the clinical record; and (5) six clinical vignettes that serve as illustrative examples to be used in training and support sessions for healthcare professionals.

### 3.2. Risk Assessment Form

The risk assessment form covers all the important points for assessing the likelihood of a suicide attempt. It is clear, precise and easy to use, since it involves answering either by checking a box or briefly indicating the presence of certain behaviors associated with suicide risk. It enables professionals to structure data collection, summarize the situation, and identify protective factors (such as social activities and leisure) that can enhance well-being and quality of life. For patients who cannot express their distress verbally, the assessment form lists behavioral and emotional changes that provide important clues for risk evaluation. As mentioned previously, the assessment does not provide a standardized categorization of the level of risk; however, participants in the pilot program frequently expressed that they would have liked to conclude their evaluation of the situation with a final rating to determine whether intervention was needed [76]. They seem to seek an objective evaluation of suicide risk, even though the predictive value of such assessments is an illusion [73].

### 3.3. Safety Plan Form

The safety plan form is a concise document that can be completed by the healthcare team and/or relatives, in collaboration with the individual with NCD if they have sufficient verbal and cognitive abilities. It mainly identifies strategies to secure the environment as well as the names of resources to contact during a crisis. It also provides a list of the person’s past and current reasons for living, which can be drawn upon in potential critical or distressing moments.

### 3.4. Vignettes

Because participants in the pilot study noted that the vignettes depicted only mild cases, the final version included scenarios depicting suicidal behavior in people with advanced NCD, as well as vignettes reflecting situations encountered in home care services [76,77]. Also, two of the vignettes were incorporated into the assessment form for training purposes.

### 3.5. Integration Workshop

Since healthcare workers are expected to have read the guide, a 3-h integration workshop briefly reviewed its main components and supporting tools, including suicide risk assessment and safety planning forms. Half of the workshop was devoted to practice, using one of the vignettes. Specific circumstances in which the Guide should be used instead of the usual protocols were also explained during this session.

Interestingly, participants in the pilot study reported that the integration workshop helped them deconstruct preconceived ideas about the low risk of suicide in individuals with NCD [76]. Most importantly, it reduced their tendency to trivialize behaviors that signal distress or suicidal intent, which are often mistakenly dismissed as mere “normal symptoms of dementia”. Participants also indicated that interactive continuous training would help consolidate their understanding of the material, especially by discussing real-life situations they had encountered and the way the Guide and its tools could be applied [76]. Finally, post-training follow-up should address recurrent suicidal ideation since participants mentioned having difficulties in intervening with individuals with this pattern. Evidently, the integration workshop should be adjusted to the professionals, teams, and geriatric units likely to use them as well as the contextual realities of the healthcare system.

## 4. Discussion

According to the pilot evaluation, the Guide met care professionals’ needs for knowledge on preventing distress and suicide in patients with NCD [76]. Nonetheless, future research should evaluate the revised version of the Guide to determine empirically whether the training and information it provides truly help to reduce suicidal behaviors among patients. Research should also be undertaken with representative samples of each professional group (e.g., nurses, social workers). It should be noted that orderlies were not invited to participate in the suicide prevention training, although several professionals emphasized that including all staff could improve the detection of distress and suicidal behavior. Future studies should assess whether involving orderlies enhances screening and prevention for people with NCD.

It should be noted that the Guide and assessment/safety plan forms are flexible tools, to be adapted as needed while respecting the core concepts and overall reflective process. At the same time, it is important to address healthcare workers’ feelings of insecurity about the quality of their assessments, particularly when it is difficult to reach a clear evaluation of suicide risk, due to challenges in obtaining information on specific indicators. Certainty is unattainable in the context of NCD. Instead, professionals must focus on reducing distress and BPSD by addressing the unmet needs of the individual with NCD, as meeting these needs may help structure safety plans and reduce suicide risk.

Overall, organizational barriers and human resources represent the main challenge professionals may face when using the Guide. In fact, older adults with NCD receive care from different professionals from various sectors of the health system, yet each works independently. Although the health and social services system aims to provide a continuum of care for older adults with NCD, many organizations are characterized by fragmented, siloed, provider-focused care [86], leading to poor coordination, duplicated efforts, barriers to timely access, and negative outcomes for the patient. Similarly, participants in the pilot evaluation reported difficulties in strengthening protective factors [76], which are largely linked to leisure and social activities often affected by budget cuts. Therefore, the lack of stimulating activities in LTC facilities somewhat discourages professionals who feel powerless in implementing them.

Finally, in Quebec (Canada), where the study was carried out, advance requests for medical assistance in dying (MAID) were only recently legalized (October 2024) for individuals diagnosed with NCD, and the law imposes strict eligibility and procedural criteria [87]. Future training programs should address this important issue, since patients might wish to end their lives before losing their decision-making capacities and their desired way of life [37]. In fact, during the three webinars on the Guide, attended by over 2,000 participants, the issue of suicide prevention in the context of MAID was frequently raised by attendees. On the CRISE website [77], a “Frequently Asked Questions” tab will be added in 2026 to explain that the wish to die, whether in suicidal individuals or those requesting MAID, is associated with major distress that should be addressed by healthcare professionals before considering death as the only solution [88]. While it can be challenging to find a balance between respecting the patient’s autonomy and alleviating their suffering, the Guide primarily focuses on addressing suicidal distress in individuals living with NCD, particularly those with moderate to advanced cognitive decline, which precludes them from making an advance MAID request.

## 5. Conclusions

The scoping review revealed that suicidality is highly prevalent among individuals with NCD, yet existing suicide risk assessment tools are not suited for this population, and interventions or care guidelines remain very scarce and largely untested. This gap underscores the urgent need for tailored approaches that consider the unique cognitive, emotional, and social challenges faced by individuals with NCD. The clinical guide that was developed during the research project draws on a scoping review of the current literature on suicide among individuals with NCD and the expertise of psychogeriatric specialists. It supports healthcare professionals in the assessment of needs and suicide risk among patients with NCDs and in the formulation of action plans to improve well-being and quality of life. By providing practical strategies, the Guide enhances patient safety but also facilitates multidisciplinary collaboration and proactive care. The pilot evaluation of the Guide was positive, with participants reporting its usefulness for their daily work and their increased confidence in recognizing and managing suicidality among patients with NCD. This increase in self-efficacy might also contribute to reducing burnout among health professionals working with this population [89]. Future research should focus on larger-scale implementation to determine the Guide’s impact on patient outcomes and long-term sustainability in geriatric settings.

## Data Availability

No new data were created or analyzed in this study.
